# The Tip of the Iceberg: Pathway Biology Must Anchor the Next Generation of Critical Care Trials

**DOI:** 10.1097/CCE.0000000000001421

**Published:** 2026-05-28

**Authors:** Logan R. Van Nynatten, Douglas D. Fraser, Tristan Look-Hong, Marat Slessarev, John Basmaji

**Affiliations:** 1 Division of Critical Care Medicine, Western University, London, ON, Canada.; 2 Division of Pediatric Critical Care Medicine, Western University, London, ON, Canada.

**Keywords:** biomarkers, clinical trial design, critical illness, omics, predictive enrichment, systems biology

## Abstract

Heterogeneity of treatment effect has yielded decades of negative critical care trials. Syndromic diagnoses like sepsis and acute respiratory distress syndrome mask distinct molecular programs that respond differently to the same intervention, and single biomarkers lack the resolution to capture this complexity. Recent evidence now demonstrates that each step of the enrichment pipeline, from real-time bedside endotyping to prospective endotype-matched therapy, is clinically operational. However, current approaches rely on limited biomarker panels that capture only surface-level biology. Pathway biology, examining coordinated dysregulation of molecular networks rather than isolated analytes, offers the deeper resolution needed to match patients to targeted therapies. We propose a translational pipeline integrating multiomics, pathway analysis, machine learning, and point-of-care assays to advance critical care toward pathway-focused predictive enrichment.

## THERAPEUTIC CRITICAL CARE TRIALS AND THE HETEROGENEITY OF TREATMENT EFFECT

For decades, critical care trials have yielded predominantly negative results (1). These shortcomings likely reflect heterogeneity of treatment effect (HTE): patients with identical syndromic diagnoses respond differently to the same intervention (2, 3). Clinical syndromes such as acute respiratory distress syndrome (ARDS), sepsis, and traumatic brain injury provide useful diagnostic frameworks, but these labels mask substantial variability in pathobiology and etiology. When investigators enroll patients solely on syndromic criteria, they combine subgroups that benefit from an intervention with those who derive no benefit or experience harm, diluting the therapeutic signal. By concentrating treatment effects within the enrolled population, predictive enrichment (PE) increases the signal-to-noise ratio and offers a transformative solution to HTE (4). This strategy identifies patients likely to respond to therapy based on underlying pathobiology. Oncology exemplifies this approach: HER2 status redefined outcomes in breast cancer via trastuzumab (4), and PD-L1 expression guides checkpoint inhibitor use in lung cancer (5). Yet critical care has struggled to adopt analogous strategies (6), partly because single biomarkers cannot capture the complexity of dysregulation in critical illness (7). Our recent systematic review demonstrated that several trials using simple biomarkers for PE have yielded negative results, in part because single measurements do not adequately represent the complexity of dysregulation in critically ill patients (6). Here, we argue that pathway-based PE—enrolling patients based on dysregulated signaling pathways rather than individual analytes—is no longer theoretical. Recent evidence demonstrates that each step of the translational pipeline, from bedside endotyping to pathobiology-matched therapy, is now feasible.

## PREDICTIVE ENRICHMENT TRIALS IN CRITICAL CARE ARE YIELDING PROMISING RESULTS

With evidence emerging at each critical step, several recent trials demonstrate that pathway-based PE has moved from concept to bedside implementation. These translational pipelines require four key steps before they can be successfully implemented at the bedside.

First, any enrichment strategy requires real-time identification of biologically meaningful subgroups. The PHIND study (Point of Care Assay to Identify Phenotypes in the Acute Respiratory Distress Syndrome) deployed the Randox multiSTAT across 30 ICUs to prospectively classify patients with acute hypoxemic respiratory failure. By measuring interleukin (IL)-6, soluble tumor necrosis factor receptor 1, and bicarbonate within 1 hour, the study identified hyperinflammatory and hypoinflammatory endotypes with distinct prognoses, demonstrating the feasibility of real-time omic endotyping (8).

Second, enrichment should also yield positive trials where unenriched approaches fail. In Tigris, patients with endotoxic septic shock (defined by Endotoxin Activity Assay) who were randomized to polymyxin B hemoadsorption had a 95.3% posterior probability of 28-day mortality benefit with intervention. Across EUPHRATES and Tigris, greater than 35,000 patients were screened without identifying this endotype clinically, underscoring its biological specificity (9, 10).

Third, enrichment must also prospectively match therapies to endotypes. ImmunoSep stratified sepsis using ferritin and monocyte human leukocyte antigen–DR (mHLA-DR) into macrophage activation-like syndrome (treated with anakinra) and immunoparalysis (treated with interferon-γ), with endotype-matched therapy improving organ dysfunction (11).

Lastly, computational approaches further refine enrichment and reveal hidden effects. A 3-biomarker model (procalcitonin, soluble triggering receptor expressed on myeloid cells 1 [sTREM-1], IL-6), validated across five cohorts, identified a hydrocortisone survival benefit in Community-Acquired Pneumonia: Evaluation of Corticosteroids (CAPE COD) limited to patients with severe immune dysregulation (12). In Efficacy, Safety and Tolerability of Nangibotide in Patients with Septic Shock (ASTONISH), nangibotide did not improve Sequential Organ Failure Assessment overall but showed benefit in patients with elevated sTREM-1, informing a phase 3 trial with prospective biomarker enrichment (13). Together, these examples define an iterative paradigm: discovery of biological states, computational refinement of enrichment criteria, and prospective validation in stratified trials.

## PATHWAY BIOLOGY AS THE UNIFYING FRAMEWORK

A common theme across these successful PE trials is the identification of specific dysregulated biological programs and the selection of therapies aligned to those underlying mechanisms. Pathway biology clarifies the molecular mechanisms that drive these dysregulated states, enabling finer patient stratification, more precise therapeutic targets, and ultimately more effective treatments.

Rather than measuring isolated molecules, pathway biology examines how genes, proteins, and metabolites operate as coordinated molecular networks, such as phosphoinositide 3-kinase/protein kinase B, MAPK/ERK, or nuclear factor kappa B (NF-κB) signaling (14–22). Disease states in critical illness arise not from the aberration of a single molecule but from coordinated dysregulation of interconnected pathways. Clinicians already recognize biological states like endothelial dysfunction, coagulopathy, hyperinflammation, and immune exhaustion, and define them using superficial markers: elevated D-dimer/international normalized ratio/partial thromboplastin time for coagulopathy, elevated IL-6 for hyperinflammation, or reduced mHLA-DR for immune exhaustion. These classifications identify that a problem exists but fail to reveal the molecular mechanisms driving it. For example, elevated IL-6 signals inflammation but does not indicate whether NF-κB, JAK-STAT, or another upstream pathway is driving it: a distinction that matters because each pathway is targetable by distinct therapeutic strategies. Pathway biology resolves this ambiguity, revealing the mechanistic architecture that surface biomarkers cannot.

Identifying dysregulated pathways requires multiple biological measurement platforms, each capturing a distinct molecular dimension. Genomics reveals inherited variation that shapes baseline host responses (23, 24). Transcriptomics captures which genes are actively expressed, reflecting dynamic response states (25–27). Proteomics quantifies the direct effectors of inflammation and organ injury, linking molecular mechanisms to clinical phenotype (28–30). Metabolomics profiles small molecules arising from cellular reactions and, because metabolites sit downstream of genetic, transcriptomic, and proteomic processes, provide real-time readouts of cellular pathway activity (31–35). A growing array of bioinformatics methodologies can map individual marker measurements onto molecular programs, revealing which pathways are dysregulated in a given patient (14–22). Integrating these omic layers reveals coordinated pathway dysregulation, producing “pathway-focused endotypes” that are more biologically precise and more likely to respond to targeted therapies.

Recent multiomic studies in sepsis demonstrate that integrating omic layers identifies robust endotypes to stratify patients. By integrating multiomic data from large multicenter cohorts, investigators identified patient endotypes with differential survival responses to specific therapies, including fluid resuscitation strategies and immunomodulator therapy in sepsis (36, 37). Routine clinical variables could not distinguish these molecularly defined subgroups. Notably, when investigators defined subgroups by differences in treatment response rather than by clustering patients with similar biology, the resulting subtypes were more consistent across omic datasets and more reproducible across cohorts. Investigators then distilled complex molecular signatures into focused proteomic panels (6 and 10 proteins, respectively) that preserved discriminatory accuracy and are amenable to rapid clinical measurement, thereby demonstrating the translational path from multiomic discovery to trial-ready stratification. Notably, these studies defined subgroups by differential treatment response rather than by pathway analysis—a complementary approach that can be used alongside or upstream of pathway-based enrichment.

## HIDDEN GRANULARITY AND THE NEXT GENERATION OF ENRICHMENT

**Figure 1.**
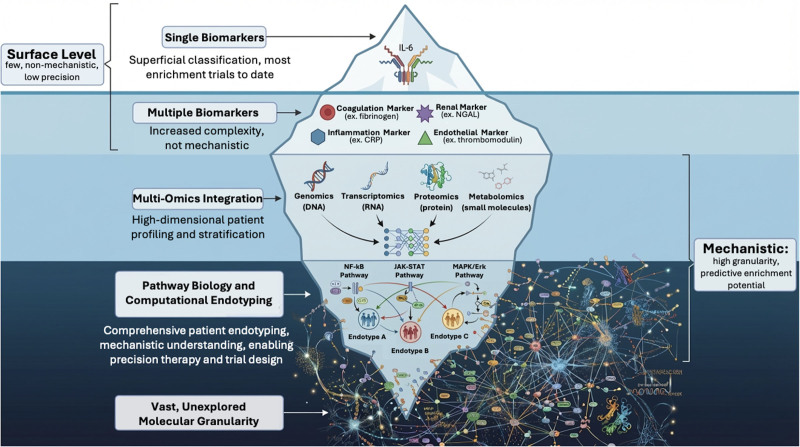
Unseen biological heterogeneity as the driver of negative clinical trials. Conceptual schematic illustrating how conventional clinical trial enrollment based on syndromic diagnoses captures only the visible “tip of the iceberg,” representing superficial clinical phenotypes and limited biomarker signals. Beneath this surface lies a large, unobserved layer of biological heterogeneity composed of diverse, coexisting molecular programs. Patients classified under the same syndrome may occupy distinct positions within this hidden biological landscape, leading to divergent responses to the same therapy. Traditional trial designs that ignore this underlying complexity dilute treatment effects by combining biologically dissimilar subgroups. In contrast, pathway-based approaches aim to resolve this hidden heterogeneity by identifying and targeting the specific molecular programs driving disease, thereby enabling biologically informed patient stratification and improving the likelihood of detecting true therapeutic benefit. CRP = C-reactive protein, IL-6 = interleukin-6, JAK-STAT = Janus kinase–signal transducer and activator of transcription, MAPK/Erk = mitogen-activated protein kinase/extracellular signal-regulated kinase, NGAL = neutrophil gelatinase-associated lipocalin, NF-κB = nuclear factor kappa B.

The aforementioned trials represent what biomarker-based enrichment achieves today. Emerging evidence reveals how much further the field can advance by fully leveraging pathway-based biology (**Fig. 1**). Within the hyperinflammatory endotype of ARDS, multiomic integration has uncovered at least three distinct molecular signatures: enhanced innate immune activation coupled with increased glycolysis, hepatic dysfunction paired with impaired fatty acid beta-oxidation, and interferon program suppression coupled with altered mitochondrial function (38). Within patients classified as hyperinflammatory, transcriptomic and proteomic endotyping has similarly identified dysregulation of innate immune responses such as tissue remodeling, zinc metabolism, collagen synthesis, and neutrophil degranulation (39). We envision an adaptive platform trial that stratifies hyperinflammatory ARDS patients by their underlying pathway dysregulation and allocates each subgroup to a matched therapeutic intervention.

Furthermore, in sepsis, effective enrichment must also account for pathogen biology. In a retrospective analysis of 8280 critically ill sepsis patients, pathogen characteristics (identity, burden, virulence, and site of infection) independently drove assignment to a hyperinflammatory endotype, with gram-negative *Enterobacterales* showing the strongest association (40). A patient may appear hyperinflammatory not because the host immune response has dysregulated but because the pathogen itself provokes an aggressive inflammatory reaction. These two scenarios likely require different therapies, yet enrichment strategies based solely on host biology cannot distinguish between them. Future sepsis trial designs should integrate host and pathogen pathway characterization to avoid this misclassification.

## OPERATIONALIZING THE PIPELINE

**Figure 2.**
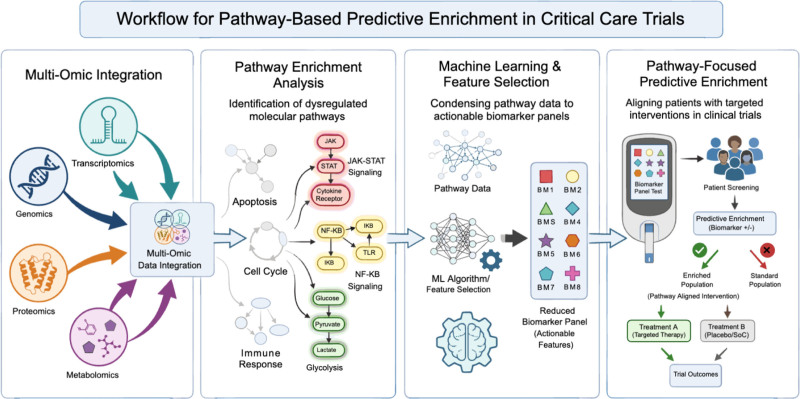
Workflow for pathway-based predictive enrichment in critical care trials. Schematic illustrating a four-step translational pipeline linking discovery multiomics to biologically informed trial enrollment. **A**, Multiomic integration: genomic, transcriptomic, proteomic, and metabolomic data are integrated into a unified systems-level dataset. **B**, Pathway enrichment analysis: integrated molecular profiles are mapped to coordinated dysregulated programs (e.g., apoptosis/cell cycle regulation, immune activation, Janus kinase–signal transducer and activator of transcription [JAK-STAT] signaling, nuclear factor kappa B [NF-κB] signaling, glycolysis), defining mechanistic endotypes. **C**, Machine learning (ML) and feature selection: computational modeling reduces complex pathway signatures into a parsimonious, clinically actionable biomarker panel. **D**, Pathway-focused predictive enrichment: the reduced biomarker panel is deployed as a rapid screening tool to identify biomarker-positive patients for targeted therapy, enhancing detection of treatment effects within biologically aligned subgroups. BM = biomarker, IκB = inhibitor of kappa B, SoC = standard of care, TLR = Toll-like receptor.

A critical barrier to biomarker-driven enrichment in critical care is that assay turnaround often does not match the therapeutic urgency. Trials require biomarker results within hours of presentation to randomize patients before the therapeutic window closes, yet most omic platforms require centralized laboratory processing with turnaround times of days to weeks. We propose a four-step translational pipeline that bridges multiomic discovery and bedside implementation (**Fig. 2**).

The first step, multiomic integration, combines genomic, transcriptomic, proteomic, and metabolomic data from critically ill cohorts to map the molecular architecture of disease. Each omic layer captures a distinct dimension of biology, and their integration reveals coordinated dysregulation invisible to any single platform. This step moves beyond the candidate-biomarker paradigm that has historically constrained critical care enrichment. Instead of testing preselected molecules, multiomic integration discovers the biological programs that distinguish patient subgroups through unbiased, data-driven analysis.

The second step, pathway analysis, maps integrated multiomic measurements onto known signaling networks to identify which molecular programs are dysregulated in a given patient or subgroup. This is the key intellectual advance over existing biomarker enrichment approaches. Rather than selecting patients by the concentration of an individual analyte, pathway enrichment analysis identifies the upstream signaling architecture producing those analyte elevations. Two patients may share elevated IL-6 yet harbor fundamentally different pathway dysregulation (e.g., one driven by NF-κB activation and the other by JAK-STAT signaling) requiring distinct therapeutic strategies. Pathway-level resolution provides a mechanistic rationale for treatment selection that isolated biomarkers cannot.

The third step, machine learning (ML) and feature selection, connects high-dimensional pathway data to clinical usability. ML identifies nonlinear relationships and multivariate interactions that conventional analyses miss, reducing hundreds of pathway-level features into parsimonious biomarker panels that retain biological and predictive signal (41–46). Unlike approaches that rely on biomarkers selected a priori or from limited datasets, this strategy derives panels directly from high-dimensional, pathway-informed data, making it more likely that selected markers reflect genuine disease biology rather than surrogate signals. Recent multiomic studies in sepsis distilled complex molecular signatures into focused panels of 6–10 proteins that preserved discriminatory accuracy and enabled rapid clinical measurement (36, 37). A potential tension warrants direct acknowledgment: if a pipeline culminates in a 6–10 protein assay, how does it differ from the small-panel enrichment strategies we critique? Traditional panels are selected a priori and read as direct thresholds, so two patients with elevated IL-6 receive the same label regardless of the pathway driving it. A feature-reduced pathway-derived panel, by contrast, is selected as the minimal set of markers that recapitulates the underlying multiomic pathway state, mapping patterns rather than thresholds onto mechanistic endotypes. Confirming pathway resolution after compression now requires prospective validation to show that the reduced panel reproduces multiomic endotypes, distinguishes overlapping analyte profiles with distinct pathway states, and preserves treatment-effect heterogeneity in clinically meaningful ways.

The fourth step, pathway-focused PE, deploys these reduced biomarker panels at the bedside to screen patients, classify their pathway dysregulation, and allocate them to targeted interventions. Rapid point-of-care measurement, whereas in its infancy, is feasible, as shown by the PHIND (8), and ImmunoSep trials (11). Furthermore, advances in mass spectrometry, microfluidic devices, and portable biosensors offer additional routes to bedside implementation (47–49). What this pipeline adds is a systematic, generalizable framework connecting each step: pathway-informed panels derived from integrated molecular data, validated through ML, and deployed at the point-of-care level to match patients to mechanism-specific therapies.

Notably, several implementation challenges warrant acknowledgment. Critical illness biology is dynamic, and endotypes assigned at enrollment may shift over hours to days, potentially requiring repeat or longitudinal profiling. Biologically specific treatable traits can also be rare in unselected ICU populations, producing unfavorable screening-to-enrollment ratio with implications for feasibility and cost. Finally, distilling high-dimensional multiomic signatures into parsimonious panels within modestly sized critical care cohorts carries a meaningful risk of overfitting, and will require rigorous external validation across independent sites and platforms, alongside prospective standardization of sample handling and analytical pipelines, to bridge the remaining gap between laboratory signature discovery and bedside-ready rapid assays. Encouragingly, each of these challenges is increasingly well defined and, with deliberate methodological and infrastructural solutions, largely addressable rather than insurmountable.

## CONCLUSIONS

Critical care stands at an inflection point. Multiomics, pathway analytics, and ML can resolve the biological heterogeneity that has long obscured treatment effects. The remaining challenge is not discovery, but implementation. Pathway-based PE provides a coherent framework to translate molecular insight into trial design, and prospective validation of these pipelines now stands as an important priority for the field. Moving forward, trials must shift from syndromic enrollment to biologically defined populations, or risk perpetuating the cycle of negative studies.
